# Leaving no one behind – understanding environmental inequality in Europe

**DOI:** 10.1186/s12940-020-00600-2

**Published:** 2020-05-27

**Authors:** Catherine Ganzleben, Aleksandra Kazmierczak

**Affiliations:** European Environment Agency, Kongens Nytorv 6, 1050 Copenhagen, Denmark

**Keywords:** Environment, Health, Well-being, Inequality, Stressors, Benefits

## Abstract

The European Union aims to deliver a healthy environment to its citizens, with significant progress achieved in tackling key environmental stressors in recent decades. At the same time, significant risks to health remain from air, soil and water pollution, noise, chemicals and the impacts of climate change. High quality environments - such as urban areas rich in green and blue spaces - offer significant benefits to health. The unequal distribution of these risks and benefits across society, whereby socially disadvantages groups are more likely to live in poorer environmental conditions, contributes to health inequity across Europe.

The European Environment Agency (EEA) is exploring how environmental risks and benefits are distributed across society. Recent evidence produced by EEA indicated that poorer European regions are more likely to be exposed to environmental health hazards at levels that negatively affect health. At country level, the disproportionate exposure of lower socio-economic groups to air pollution, noise and high temperatures is seen in urban areas. We also see inequality in terms of who generates pollution and who suffers the consequences. While poorer countries are likely to be worse affected by climate change, high incomes are linked to high carbon footprints across Europe. Quality environments offer benefits to health, in particular in urban areas, and can contribute to reducing health inequalities. Environmental inequality also plays out across generations, whereby future generations will have to tackle environmental degradation resulting from the activities of past and current populations, such as the accumulation of persistent chemicals in the environment, biodiversity loss and climate change.

New opportunities exist to explore the complex linkages between environmental quality, socio-economic status, and health and well-being. These include combining existing data from across these domains with data from new sources, such as citizen science initiatives, smart phones, social media and satellite observation data. Knowledge that integrates the social and environmental domains and explores the drivers behind environmental health inequity is crucial to supporting implementation of the United Nations (UN) 2030 Agenda for Sustainable Development, in particular the pledge of leaving no one behind.

## Background

Good health is closely connected to the state of our environment. The new agenda of the European Commission, the European Green Deal, sets the ambition of zero-pollution, to be delivered through a cross-cutting strategy to protect citizens’ health from environmental degradation and pollution. At the same time, the agenda calls for a just transition towards a green economy that leaves nobody behind [1].

The European Union (EU) aims to deliver “a high level of protection and improvement of the quality of the environment” [2], through the implementation of policies on air, soil and water pollution, environmental noise, chemicals and climate change. Over the past three decades, the EU environmental acquis has delivered significant benefits for the health and well-being of the public [3]. However, is the EU securing a consistent level of environmental quality for *all* of its citizens, or are some being left behind?

Environmental monitoring data managed by the European Environment Agency (EEA) reveals that substantial proportions of the Union’s urban population remain exposed to levels of air pollution [4] and noise [5] that exceed World Health Organisation (WHO) health-based guidance values. Citizens’ health is also adversely affected by climate change, through heatwaves and floods of increasing frequency and magnitude, as well as changes in the distribution of vector-borne diseases [6]. At the same time, European citizens do not enjoy equal access to high quality environments, which offer significant benefits to health and wellbeing.

An emerging body of evidence highlights the linkages between socioeconomic conditions and poor quality environments at both local and regional scales across the European region [7, 8]. An individual’s socio-economic status influences their exposure and their sensitivity to environmental risks, as well as their resilience in adapting to or avoiding future risks. Thus, environmental risks disproportionally affect socially disadvantaged and vulnerable population groups, exacerbating existing inequalities. WHO identifies decent living conditions as one of the five essential conditions required to sustain a healthy life. A lack of green space, poor air quality, fuel deprivation and housing deprivation are amongst the dimensions of living conditions that drive health inequities in Europe [9].

The EEA’s mandate is to produce sound, reliable and relevant information on the environment to support the European policy agenda and to inform the public. As such, the EEA is exploring the interplay of social, economic and environmental factors, investigating how environmental risks are distributed across society and assessing what this implies for public health. Another dimension under scrutiny is how social status can influence access to the benefits clean environments offer for health and well-being. This paper captures reflections that have emerged from discussions of the EEA's Scientific Committee on environmental justice in Europe, future knowledge needs and implications for environmental policies.

## What is environmental inequality?

Environmental inequality results from the unequal distribution of the risks and benefits that stem from interactions with our environment. There are several dimensions of environmental inequality where the EEA produces relevant knowledge, explored in turn below.

### The unequal distribution of environmental risks across European society

The quality of the environment varies significantly across Europe; in general terms between east and west, but also between countries, regions and neighbourhoods within cities. Whether you benefit from a high quality local environment or suffer the impacts of pollution depends on where you live and work. Recognising the negative impact of pollution on health, the EEA is exploring how the spatial distribution of pollution relates to socio-economic factors.

A recent EEA report [7] assessed the links between socio-demographic inequalities and exposure to air pollution, noise and high temperatures at various spatial scales in Europe. It went on to explore how exposure to these environmental health hazards and their health impacts are differentiated between socio-economic and demographic groups. The report concluded that the poorer European regions tend to be more exposed to environmental health hazards, often experiencing cumulative exposure to multiple environmental stressors. For example, Eastern and South-Eastern Europe experiences higher exposure to particulate matter compared to the West. In most settings, groups of lower socio-economic status tend to be more negatively affected by environmental health hazards, as a result of both higher exposure and increased vulnerability. In many European countries, the disproportionate exposure of lower socio-economic groups to air pollution, noise and high temperatures occurs in urban areas. Further, Europe-wide, elderly, children and those in poor health tend to be more adversely affected by environmental health hazards than the general population. For example, we see higher mortality rates amongst the elderly during heatwaves compared to general population. However, the quality of the available evidence varies – both in geographical terms, evidence of environmental inequalities is more extensive in Western Europe, and for different environmental health hazards, with the least knowledge on the health impacts of noise and the interplay between various hazards.

### The unequal distribution of environmental risks across generations

A second dimension in the distribution of environmental risks is temporal, whereby future generations face the risks created by the polluting activities of today. Environmental risks for which intergenerational inequities are particularly relevant include chemical pollution and climate change.

The current trend of increases in both the diversity and volume of synthetic chemicals in use is foreseen to continue, suggesting that chemical emissions will also increase. Persistent chemicals remain in the environment, in some cases bioaccumulating in food chains, for future generations to tackle as a legacy of current production and consumption patterns. Exposure to developmentally toxic chemicals during pregnancy can damage the development and future functioning of the endocrine (hormonal) system, the immune system or the neurological system (affecting brain development) of the unborn foetus [10]. EEA is working with partners under the European Human Biomonitoring Initiative, HBM4EU,^1^ to better understand human exposure to chemicals and the resulting impacts on health.

In the context of a transition to a circular economy, the recycling of products containing hazardous chemicals can jeopardise the quality of future material flows. EEA is consolidating knowledge on emerging chemicals that show persistent properties to inform policy efforts to minimise chemical pollution, as well as working with partners to promote production approaches that are safe and sustainable by design as a solution for cleaning up material flows.

With regards to climate change, the likelihood of pervasive and irreversible impacts is increasing as emissions of greenhouse gas continue, with the cost of past and present emissions to be paid by the youth of today. The EEA supports current mitigation and adaptation efforts, the evaluation of EU policies and the development of long-term strategies both to mitigate and adapt to climate change. EEA produces European level information on greenhouse gas emissions trends and projections, in support of forward looking policy actions aimed at protecting current and future generations. EEA’s assessments also explore the current efforts in Europe to adapt to future climate change, in various settings (e.g. urban areas) and sectors (e.g. transport, energy and agriculture).

### Who pollutes? Who pays?

Inequality in terms of who generates pollution and who suffers the consequences occurs at multiple levels and is a driver of social inequity. Research to date has focused on carbon inequity, with the wealthiest 10% of the global population responsible for around 45% of global emissions [11]. A recent analysis of EU countries found incomes to be the most important driver of a region’s carbon footprint [12]. At the same time, the poorer countries are likely to be more affected by climate change-related weather extremes, both globally [13] and within Europe [14].

In terms of the EU’s impact beyond its borders, the EEA measures the ecological footprint for Europe, tracking the area of biologically productive land and water required to produce all the biological resources that European society consumes and to absorb the waste that we generate. The EU-28 region’s footprint is over twice the size of its capacity to produce useful biological materials and to absorb waste materials [15]. As such, much of the impact of EU consumption manifests in environments outside the EU, with implications for the health of populations inhabiting those environments.

### Unequal access to environmental goods

Uneven access to high quality environmental goods is another dimension of environmental inequality. Nature offers public health benefits in terms of improved mental health, and reductions in cardiovascular disease, obesity and type 2 diabetes, as well as improved pregnancy outcomes [16]. Improved access to green space has been shown to reduce health inequalities in urban areas and to contribute to social cohesion [17].

Work has be already been undertaken by the European Commission using Copernicus Urban Atlas data to assess the spatial distributions of populations and green areas in urban areas [18]. Opportunities exist for EEA to assess how socio-economic status might affect access to green space in urban areas and quality environments outside cities, such as national parks, by mapping data provided under the Copernicus Land Monitoring Service against available socio-economic data at different scales.

### Access to procedural justice

Procedural justice implies that different groups have equal access to decision-making and justice regarding their environments. The Aarhus Convention [19] provides for access to environmental information, participation in decision-making and access to justice. In this context, the EEA provides information to the public about local, national and European environmental quality and how this may affect health.

As an example, public awareness of poor air quality plays a critical role in generating pressure to tackle air pollution. Recently, citizens have been getting more involved in air quality issues and have gone to national Courts, which in several Member States ruled in favour of their right to clean air [20].

## New knowledge to better understand environmental inequality in Europe

Environmental inequality issues sit at the nexus of environment, society and economy. Innovative analysis in this field requires the combination of different types of data, including data on socio-economic status, health and local environmental quality. Implementing such approaches may require new partnerships between organisations operating in separate domains, such as health, environment, and urban planning, and at different scales, such as European, national and city level. A challenge is to identify datasets for the same scale and timeframe that can be meaningfully combined. Combining data on environmental quality with both social and health data at various spatial scales could yield valuable insights regarding associations between exposure, social status and health outcomes. At the same time, harnessing health data and interpreting it at the level of the individual is sensitive, and compliance with the General Data Protection Regulation [21] presents an immediate challenge.

The linkages between exposure, vulnerability and sensitivity to environmental stressors are complex and relate to the social circumstances, health, behaviours and genetic profile of each individual. In terms of the design of studies intended to explore this nexus, cohort studies are well suited to exploring the roles that exposure to environmental risks and access to environmental benefits play as health determinants along the lifespan of an individual. Innovative tools and methods are being deployed to measure environmental exposures at an individual level. Smart phones already include ambient light meters, Global Positioning System sensors, and accelerometers, which measure movement and can be used to determine travel models. This data on location and movement can be combined with data on environmental quality produced by small sensors worn by individuals, as well as data on each individual’s socio-economic status and behaviour. While not directly involved in research activities, EEA readily harvests results emerging from the scientific community and translates this evidence into key messages for the policy making community.

New opportunities exist for understanding the quality of local environments through citizen science, whereby the public contributes to data gathering. The EEA is currently working collaboratively with the European Network of the Heads of Environment Protection Agencies to gather information on children’s exposure to air pollution in schools, using low cost sensors put in place by the school authorities and involving school children. At the same time, the data generated by citizen science poses challenges in terms of robustness and spatial coverage, with further reflection required on how it might best be combined with the results of coordinated monitoring activities under the Air Quality Directive [22]. The HBM4EU project provides another example where scientists are taking samples from citizens to produce knowledge on their chemical body burden - a very personal type of pollution. Such approaches have the potential to democratize environmental information and actively engage the public in implementing solutions at local level, including making changes to their own behaviour.

One approach to connecting with citizens is to demonstrate how their personal health is linked to the quality of their local, national and global environment. This entails packaging information for public consumption, using non-technical language and distilling key messages. An example of a useful tool is the EEA Air Quality Index,^2^ which allows citizens to compare air quality across Europe in real time and includes concise messages on health impacts.

Environmental inequality is a driver of health inequity, fostering feelings of injustice and being “left behind” amongst vulnerable populations. Public discourse on environmental inequalities should respond to protests against socio-economic inequalities and widespread dissatisfaction with political inaction in tackling climate change. In order to do so, knowledge on environmental inequalities should be communicated using accessible language, tailored to specific audiences and released at key moments. Such communication products can make use of new forms of data presentation, such as maps overlaying different types of information and infographics explaining exposure pathways. As mentioned above, there are opportunities to exploit Copernicus data and use rapidly evolving data visualisation tools to provide compelling web-based insights on environmental inequity across Europe. Combining macro-scale data on land use and ecosystem quality with micro-scale data on individual health has the potential to reveal associations.

## Reflections on the policy landscape

In recent years, global frameworks and agreements have captured the concept of environmental equity. Inclusion is at the core of the United Nations (UN) 2030 Agenda for Sustainable Development [23], reflected in the pledge to leave no one behind and in the vision of a “just, equitable, tolerant, open and socially inclusive world in which the needs of the most vulnerable are met”. The Office of the UN High Commissioner for Human Rights recently published Framework Principles on Human Rights and the Environment [24], which clarify the human rights obligations of States relating to safe, clean, healthy and sustainable environments. These include ensuring protection against discrimination in relation to the enjoyment of such environments, the delivery of non-discriminatory environmental standards, measures to protect the rights of those vulnerable to environmental risks and public participation in environmental decision-making. The 2015 Paris Agreement on Climate Change [25] emphasises the importance of considering the rights of vulnerable people when taking action to address climate change.

At the pan-European level, the European Environment and Health Process brings together policymakers from the health and the environment domains to shape relevant policies and actions. Agreed in 2017, the Ostrava Declaration summarises the priorities in these areas in the WHO European Region, resolving to protect and promote health and well-being and prevent premature deaths, diseases and inequalities related to environmental pollution and degradation [26].

At European level, the European Commission recognises that the health of the planet and of citizens go together and has launched the European Green Deal [1]. This sets ambitious goals for Europe to become the first climate-neutral continent, to move towards zero-pollution and to be a world leader in implementing a circular economy. At the same time, the transitions required to deliver the European Green Deal should be just, recognising that people start from different points and that some require tailored support. These bold ambitions reach across the social, environment and health domains and will require integrated approaches based on cross-cutting knowledge.

While the EU has some of the world’s most progressive policies for delivering social equity to European citizens, delivering *environmental equity* has thus far not been an explicit goal. Nevertheless, the link between the social, environment and health dimensions is touched upon in recent policy cross cutting frameworks. The European Commission’s 2019 reflection paper “Towards a sustainable Europe” [27] emphasises that the ecological transition to sustainability must be socially fair, delivering benefits to health and social well-being across European society and leaving no one behind. The Urban Agenda for Europe acknowledges the structural dimensions of poverty in deprived urban neighbourhoods and calls for integrated approaches to urban regeneration, with a focus on air pollution and the social dimension of climate adaptation strategies [28]. The recent evaluation of the EU Adaptation Strategy highlights the areas where the strategy may be able to deliver more in the future, including a focus on social vulnerability in adaptation policies and more explicit links between health and climate change [29]. The EU Green Infrastructure Strategy emphasises the role of green spaces in urban areas in building communities and combating social exclusion [30].

Policies to deliver environmental equity should aim at preventing and reducing the socio-spatial concentration of environmental health risks, ensuring fair access to environmental resources and enabling sustainable choices. As of today, the social distribution of environmental risks and indeed, the benefits associated with high quality environments, is rarely tackled by EU thematic policies aimed at improving the quality of environmental media. A first step would be to explicitly integrate these goals into existing environmental legislation, such as the agendas for climate change, noise and air pollution. In order to address the unequal social distribution of environmental stressors, universal efforts to deliver overall reductions in exposure for the general population can be complemented by measures targeted at groups known to be vulnerable in terms of their higher levels of exposure, increased sensitivity or reduced resilience. Following the principle of “proportionate universalism”, in order to reduce the steepness of the social gradient in health, actions must be universal, but with a scale and intensity that is proportionate to the level of disadvantage [31]. Efforts to reduce inequalities in exposure should focus on bringing the exposure of all groups to levels at which impacts on health are considered negligible according to current scientific knowledge.

A second step would be to integrate the concept of environmental inequity into other policy domains, such as policies addressing the determinants of diseases or policies targeting urban planning. The Urban Agenda for the EU [28] seeks to improve the quality of life in urban areas and to contribute to smart, sustainable and inclusive growth. Policy actions in one domain have the potential to offer co-benefits across other policy domains. For example, measures to promote active transport in deprived neighbourhoods can increase physical activity while also reducing vehicular emissions and improving air quality. Campaigns to promote low meat diets can yield improvements in cardiovascular health, as well as reducing emissions of greenhouse gases [32]. Improving access to quality green and blue spaces in urban environments can deliver a triple win, including improved health outcomes, social cohesion and mitigation of environmental stressors, such as noise and extreme heat [33].

At European level, options exist to target socio-environmental inequalities through EU cohesion funds, as environmental inequalities seem to follow the pattern of socio-demographic inequalities across Europe. This could be achieved through developing specific funding aimed at improving environmental quality in disadvantaged urban neighbourhoods, as well as at enabling people to make more sustainable choices, such as switching to cleaner fuels.

At the national and local level, multiple policy areas from welfare policies to urban design can help to reduce the exposure of vulnerable populations to environmental stressors. The exact policy levers depend on the hazards and types of vulnerable populations that require addressing. For example: heat wave action plans involving health and social care sectors are an effective method of reducing mortality amongst the elderly during hot spells; building design and spatial planning can ensure that schools are located and built in a way that does not expose children to road traffic noise; and low emission zones in cities can improve air quality in city centres occupied by disadvantaged communities [7]. Effective communication across different levels of governance when planning actions to address inequalities in exposure to environmental stressors can support the implementation of a coordinated approach.

## Conclusions

In a context where the environment is a public good, the fact that poorer people live in more degraded environments and suffer worse health impacts, compounds other concerns about fairness and trust in our society. Europe would benefit in public health, economic and broader societal terms from integration of environmental equality into EU policies, ensuring that the goal of delivering a high quality environment is met for all European citizens.

By producing Europe-wide, accessible knowledge on how the environment affects health, EEA is in a position to bring the social and environmental agendas together and support the European Green Deal. Providing information that is both relevant for decision makers and that resonates with the general public can motivate citizens to engage with their local environments and push for improvements through an inclusive policy agenda.

## Data Availability

Not applicable.

## Abbreviations

EEA: European Environment Agency
EU: European Union
UN: United Nations
WHO: World Health Organization

## Footnotes

1 www.hbm4eu.eu

2 http://airindex.eea.europa.eu/
